# A comparison of quality of life between HIV positive and negative diamond miners in South Africa

**DOI:** 10.1080/17290376.2013.870066

**Published:** 2014-01-03

**Authors:** Jeff Gow, Gavin George, Kaymarlin Govender

**Affiliations:** a PhD, Associate Professor, is affiliated to the School of Accounting, Economics and Finance, University of Southern Queensland, Toowoomba, Australia; b PhD, Research Associate, is affiliated to the Health Economics and HIV and AIDS Research Division (HEARD), University of KwaZulu-Natal, Durban, South Africa; c PhD, Senior Researcher, is affiliated to the HEARD, University of KwaZulu-Natal, Durban, South Africa; d PhD, Senior Lecturer, is affiliated to the School of Psychology, University of KwaZulu-Natal, Durban, South Africa

**Keywords:** health-related quality of life, South Africa, miners, Qualité de vie sanitaire, Afrique du Sud, mineurs

## Abstract

**Objective:**

To analyse the health-related quality of life (HR-QOL) in two groups of diamond miners (HIV negative and positive) in South Africa using three instruments. Two hypotheses were to be tested. One, was that the HR-QOL of HIV positive miners would be lower than that of HIV negative miners; and two, the selected instruments would behave consistently and thus all would confirm hypothesis one.

**Methods:**

In our study, workers were recruited during a voluntary counselling and testing programme for HIV. HR-QOL were estimated using the Assessment of Quality of Life (AQOL) Mark 2, EQ-5D (EuroQOL), and Health Utilities Index 3 (HUI3) instruments. The data were analysed for utility values and for correlations between variables of interest (in particular HIV status). Goodness of fit, Pearson's *r* coefficient and *t*-tests were the statistical tests applied to the data.

**Results:**

Just over 1100 respondents were included in the analysis. HIV positive workers scored significantly lower on quality of life on the HUI3 as compared to HIV negative workers but this relationship did not (surprisingly) hold for the AQOL or EQ-5D. There was a significant positive correlation between all three instruments.

**Conclusion:**

There was inconsistency among the instruments in measuring quality of life differences according to HIV status. The HUI3 confirmed the a priori expectation that the HR-QOL of HIV positive miners would be lower than HIV negative miners. There was no statistical difference for the AQOL and a confounding result was found for the EQ5D.

## Abstract (FR)

**Objectif:**

Analyser la qualité de vie sanitaire (HR-QOL) auprès de deux groupes de mineurs de diamant (Séropositifs et séronegatifs) en Afrique du Sud en utilisant trois outils. Deux hypotheses devaient être testées. La première était que le HR-QOL des mineurs séropositifs serait inférieur à celui des mineurs séronégatifs; et la deuxième était que les outils sélectionnés se comporteraient de façon régulière et confirmeraient donc l'hypothèse un.

**Méthodes:**

Dans notre étude, les travailleurs furent recrutés lors d'un programme volontaire de soutien et de dépistage du VIH. Les HR-QOL étaient censés utiliser les outils AQOL (Evaluation de la Qualité de Vie) Mark 2, EQ-5D (EuroQOL) et HUI3 (Indice des Services Sanitaires). Les données furent analysées pour des valeurs de service et pour des corrélations entre les variables d'intérêt (en particulier le statut VIH). Les tests statistiques appliqués aux données furent le Test de l'Ajustement, le Coefficient de Corrélation de Pearson r et les tests t.

**Résultats:**

Un peu plus de 1100 personnes interrogéees furent inclues dans cette analyse. Les travailleurs séropositifs ont marqués beaucoup moins de points en qualité de vie sur le HUI3 comparé aux travailleurs séronégatifs, mais ce rapport n'a pas tenu (étonnamment) pour l'AQOL ou l'EQ-5D. Il y avait une importante corrélation positive entre les trois outils.

**Conclusion:**

Il y avait un manque de cohérence parmi les outils en ce qui concerne le mesurage des différences de qualité de vie selon le statut VIH. Le HUI3 a confirmé la prévision a priori que le HR-QOL des mineurs séropositifs serait inferieur à celui des mineurs séronegatifs. Il n'y avait pas de différence statistique pour l'AQOL et le résultat du EQ5D était déconcertant.

## Introduction and aims

The health-related quality of life (HR-QOL) of HIV positive individuals is hypothesised to be lower than for individuals living without HIV. Many studies have established this relationship in the African and elsewhere contexts (Abera, Gedif, Engidawork & Gebre-Mariam 2010; Adewuya *et al.* 2008; Beard, Feeley & Rosen 2009; Carabin *et al.* 2008; Clayson *et al.* 2006; Honiden *et al.* 2006; Hughes *et al.* 2004; Jelsma *et al.* 2005; Jia *et al.* 2007; McInerney *et al.* 2008; O'Keefe & Wood 1996; Peltzer & Phaswana-Mafuya 2008; Tsasis 2000).

We assessed HR-QOL using validated instruments in two cohorts of diamond miners. Respondents assessed their quality of life regarding their current health, as part of an integrated health intervention, including voluntary counselling and testing (VCT) for HIV. QOL

This paper reports on the HR-QOL of workers at a South African diamond mine. Two hypotheses were to be tested. One, was that the HR-QOL of HIV positive miners would be lower than that of HIV negative miners; and two, the selected instruments would be consistent and all tested instruments would confirm hypothesis one.

## Literature review

### Health related quality of life

The multidimensional concept of HR-QOL refers to how a person feels about their life and how well they function in their daily activities (Lorenz *et al.* 2001). The variety of HR-QOL instruments available reflects the lack of consensus on the number of items which should be included and how the construct of HR-QOL should be measured (Friend – du Preez & Peltzer 2009; Robinson 2004; Stavem, Froland & Hellum 2005). Only a handful of studies have looked at the QOL of people living with HIV (PLHIV) in southern Africa (Friend – du Preez & Peltzer 2009; Hughes *et al.* 2004; Jelsma *et al.* 2005; Louwagie *et al.* 2007; Peltzer & Phaswana-Mafuya 2008; Phaladze *et al.* 2005; Wouters *et al.* 2007).

### HIV, mining and HR-QOL

Numerous studies have examined the impacts of HIV in mining workplaces or communities in South Africa (Auvert *et al.* 2001; Bhagwanjee, Petersen, Akintola & George 2008; Campbell & Williams 1999; Gebrekristos, Resch, Zuma & Lurie 2005; Rispel, Peltzer, Nkomo & Molomo 2010; Stevens, Apostolellis, Napier, Scott & Gresak 2006). Three studies examined this link in other African countries (Desmond *et al.* 2005; Kis 2007; Lightfoot, Maree & Ananias 2009), and outside of Africa there have been a further three studies (Faas, Rodriguez-Acosta & Echeverria de Perez 1999; Linhares & Mello 1989; Zhang *et al.* 2007). However, literature searches did not reveal any study which linked those two issues (mining and HIV) together with measures of the quality of life of miners or members of their communities.

### Instruments to measure HR-QOL

The best choice of an instrument for the estimation of HR-QOL is not definitive (Carabin *et al.* 2008; Clayson *et al.* 2006; Coons *et al.* 2000; Hawthorne, Richardson & Day 2003; Holland *et al.* 2004; O'Brien *et al.* 2010). Generic HR-QOL instruments are designed to be applicable across a wide range of populations and interventions. Specific HR-QOL measures are designed to be relevant to particular interventions or in certain subpopulations (e.g. individuals with rheumatoid arthritis). More and more attention is being paid by policy-makers towards the use of HR-QOL, especially in associated clinical trials, given the influence that the results of these studies can have on policy outcomes and positions.

Coons *et al.* (2000) in an earlier review of generic instruments found that hundreds of generic and specific HR-QOL instruments have been developed, QOL used and/or cited in the literature. The six characteristics of an instrument addressed by the review were: (i) conceptual and measurement model; (ii) reliability; (iii) validity; (iv) respondent and administrative burden; (v) alternative forms; and (vi) cultural and language adaptations. Of the instruments reviewed, the Short Form 36 health survey (SF-36) was the most commonly used HR-QOL measure. The Health Utilities Index (HUI) and Euro QOL (EQ-5D) are preference-based measures designed to summarise HR-QOL in a single number ranging from 0 to 1. In this study, it was found that there are no uniformly ‘worst’ or ‘best’ performing instruments. The decision to use one over another, to use a combination of two or more, to use a profile and/or a preference-based measure or to use a generic measure along with a targeted measure will be driven by the purpose of the measurement.

Clayson *et al.* (2006) reviewed the existing HR-QOL measures reported in the HIV and AIDS literature since 1990. They undertook a comprehensive review following predefined selection criteria. Generic and HIV-targeted measures were assessed for content and practicality in a clinical trial setting. The generic measures were additionally reviewed for their ability to produce preference-based index scores. Three generic and six HIV-targeted measures met the selection criteria and were then assessed thoroughly in terms of their development (HIV-targeted measures), psychometric properties and appropriateness for use in clinical trials.

Although there is no one best HR-QOL measure for use in HIV and AIDS clinical trials, based on the review criteria they identified three generic and two HIV-targeted candidate measures. It was determined that each of the selected generic measures (i.e. SF-36, EQ-5D, HUI) could serve as a useful adjunct to an HIV-targeted measure in a trial. The Functional Assessment of HIV Infection and Medical Outcomes Study-HIV health survey were deemed as the two most appropriate HIV-targeted measures. Each of the measures can be self-administered in 10 minutes or less and there was evidence of their excellent psychometric properties. However, these measures have their limitations and it is clear that greater consensus needs to develop regarding more effective and efficient approaches to HR-QOL measurement in HIV/AIDS clinical trials.

Carabin *et al.* (2008) in their literature review found that several HR-QOL instruments have been developed and utilised in HIV and AIDS research. They reviewed 14 of these instruments for their accuracy in measuring construct validity, criterion validity, internal consistency reliability, sensitivity to change, and test–retest reliability.

In their definitive literature search of HR-QOL, O'Brien *et al.* (2010) did not find one instrument which was developed specifically for the purposes of describing the health-related effects of HIV.

### Selected instruments

Three commonly used preference-based instruments for measuring HR-QOL were selected for estimation purposes: These were: EQ-5D, HUI 3, and Assessment of Quality of Life (AQOL) Mark 2.

The workplace health questionnaire used for data collection purposes commenced with questions on the demographic profile of the respondent and then proceeded to ask the questions which make up the three instruments.

### AQOL 2

The AQOL instrument is a multi-attribute utility measure for use in economic evaluation, measuring HR-QOL (Hawthorne, Richardson & Osborne 1999; Richardson *et al.* 2007a, 2007b). The descriptive system can be used to provide health profiles. The AQOL instrument consists of five domains with four different levels in each; illness, independent living, social relationships, physical wellbeing and psychological wellbeing. The AQOL instrument has been revised and the AQOL 2 has included an additional dimension and item response levels to increase instrument sensitivity.

### EQ-5D

The EQ-5D is a second utility instrument for use as a measure of health outcome. Applicable to a wide range of health conditions and treatments, it provides a simple descriptive profile and a single index value for health status (Dolan 1997; Krabbe & Weijnen 2003; Kudel *et al.* 2006; Ravens-Sieberer *et al.* 2010; Szende *et al.* 2007). EQ-5D was originally designed to complement other instruments such as the SF-36, Nottingham Health Profile, Sickness Impact Profile, or disease-specific questionnaires but is now increasingly used as a ‘stand-alone’ measure. The EQ-5D instrument consists of five domains: mobility, self-care, usual activities, pain/discomfort, and anxiety/depression. Each domain is defined by three levels of function from poor to good. The three levels of domain correspond to different scores.

### HUI 3

The Health Utilities Index 3 (HUI 3) is a third generic health status and HR-QOL measure (Furlong *et al.* 1998). Its eight dimensions provide a more sensitive descriptive system than the EQ-5D but it has fewer health states than AQOL 2. In common with the AQOL 2, it uses a multiplicative scoring system which has considerable advantages over the simpler additive model used by the EQ-5D. HUI 3 has eight attributes (vision, hearing, speech, ambulation, dexterity, emotion, cognition, and pain) with five to six levels per attribute. A multiplicative multi-attribute utility function and single-attribute utility functions for HUI 3 enable users to generate utility scores for HUI 3 health states.

## Methods

This HR-QOL study was undertaken in conjunction with a VCT campaign for HIV/AIDS at a diamond mine in South Africa over a one week period in late 2008.

### Sample and study design

Only volunteers participated in the VCT and HR-QOL survey. Individuals participated in VCT only if they were convinced that they will not be discriminated against by the company, fellow employees and community members and if they stand to gain by acquiring access to medical care related to HIV and AIDS. The company provides free antiretroviral treatment (ART) to employees and their spouse or life partner, under specific conditions, including that testing will only take place where a doctor has recommended testing for medical reasons, testing will take place within a health care setting, testing must include pre and post-test counselling, VCT takes place in strict confidentiality and was conducted in line with the Department of Health's National Policy on VCT. During the time between supplying saliva for their HIV test and the results being available, the HR-QOL survey was completed under the direction of the researchers who were on hand to assist with any problems. At the mine, management and the local leadership of the National Union of Mineworkers (NUM) branch took the test to officially launch the VCT programme.

The workplace health questionnaire was given ethical approval by the Faculty of Health Sciences Ethics Committee at the University of KwaZulu-Natal, Durban. Permission to conduct the research was negotiated with company management, the local NUM shop stewards forum and the clinical staff at the study site. Informed consent was obtained from all research participants in the study and their anonymity was assured.

### Data collection

The data collection effort was coordinated with the VCT campaign at the mine. Extensive discussions with the VCT coordinator suggested that the workplace health questionnaire could be successfully streamed into the VCT campaign. To this end, the researchers provided extensive training to the VCT service provider staff, staff supervisors and others involved in facilitating the VCT programme, in the execution of the questionnaire. As part of this process, the questionnaire was pilot tested on this same population for consistency and ease of understanding. All of the survey instruments were originally in English. The three instruments which made up the questionnaire were translated into the two other most common languages in use at the mine: Afrikaans and Setswana. The translation occurred as significant numbers of the participants did not have English as their first language. The instruments were then back translated by other pilot participants to ensure consistency as recommended (Bowden & Fox-Rushby 2003). Participants were free to choose in which language they answered the questionnaire.

The VCT service provider in 2008 was a local NGO. Group VCT sessions and individual post-test counselling sessions were held in the workplace – the latter in suitable venues for privacy purposes. The counsellors were a combination of lay counsellors and professional registered nurses. The VCT sessions in the workplace usually took place at the start of a shift. All shifts were seen so coverage was 100% of the workforce.

Participation in the VCT campaign was high. A total of 1176 (83%) of permanent and temporary staff and 1605 (90%) of contractors participated in VCT. Respectively, 14% and 8% refused testing when asked. The participation rate has increased and improved since VCT commenced at the mine in 2002.

Just under 1200 participants completed the HR-QOL survey in total. That is, just over half of the VCT participants did not wish to engage with the HR-QOL survey. As all activities on the day were voluntary, the rights of participants to refuse to cooperate with the survey were respected and not questioned. The characteristics of the participants who completed the HR-QOL survey are outlined in Table 1. The frequency of missing values among the instruments was different (18% for AQOL and 15% for EQ-5D and HUI3). All missing values were ignored for estimation purposes.

**Table 1.** Demographic characteristics of participants

	*N*	%
*Gender		
Male	871	75.7
Female	118	10.3
Missing	162	14.1
*Employment status		
Permanent	402	34.9
Temporary	94	8.2
Contractor	512	44.5
Missing	143	12.4
*Race		
Black	640	55.6
Asian	16	1.4
White	178	15.5
Coloured	160	13.9
Missing	157	13.6
*HIV status		
Positive	47	4.1
Negative	1103	95.8
Missing	1	0.1
*Nationality		
South African	941	81.8
Non South African	56	4.9
Missing	154	13.4
*Job band		
A (unskilled)	105	9.1
B (semi-skilled)	218	18.9
C (artisan)	165	14.3
D/E (management)	59	5.1
Missing	604	52.5

* *p* < 0.01.

### Data analysis

The quality of life data were analysed using SPSS Version 17.0. The initial analyses included an inspection of descriptive statistics related to demographic data, including goodness of fit tests for discrete distributions for gender, employment status, race, HIV status, nationality, and job band. Pearson's r coefficients were used to examine associations among EQ-5D (EuroQOL), HUI 3 and AQOL Mark 2 measures. *T*-tests were used to examine differences in AQOL measures as a function of HIV status. For all inferential analyses, alpha was set at *α* = 0.05.

## Results

Just under 1200 miners participated in the study. The demographic characteristics of the group are described in Table 1. Variations in sample size for scales were as a result of missing values on individual questions. The data indicate that the sample was overwhelmingly male, black, HIV negative and South African.

The number of useable observations varied by instrument type, with the shortest instrument, the EQ-5D, with only five questions, having the most observations. The longest instrument, the AQOL which has 20 questions over 5 domains, had the least number of valid instruments completed successfully.

It was found that for HIV + workers the mean quality of life value was less than one, with one being perfect health (indicated by 10 out of 47 people) with observed average utility values of 0.597 (AQOL); 0.918 (EQ-5D); and 0.821 (HUI-3), respectively. The standard deviation of the three utility instruments were quite high, at 0.398 (AQOL); 0.144 (EQ-5D); and 0.324 (HUI-3), respectively. This was especially true for AQOL and HUI-3, which reduces the confidence that should be placed in the mean estimates being representative of the HIV+ cohort as a whole. An alternative measure of quality of life used in the EQ-5D is the Visual Analogue Measure (VAM) out of 100 which had a mean of 89.98 with a standard deviation of 13.85.

It was found that for HIV-workers, the mean quality of life value was less than one, with mean utility values of 0.627 (AQOL); 0.915 (EQ-5D); and 0.913 (HUI-3), respectively. The standard deviation of the three utility instruments were 0.349 (AQOL); 0.197 (EQ-5D); and 0.169 (HUI-3), respectively. The VAM had a mean of 89.13 with a standard deviation of 13.32. The standard deviation of two of the utility instruments was, however, quite low, which is unsurprising given the large sample numbers of approximately 1000 observations for each instrument, 990 (AQOL); 1016 (EQ-5D); and 1060 (HUI-3), respectively. This increases the confidence that should be placed in the mean estimates being representative of the HIV-cohort as a whole. However, the standard deviation of AQOL is surprisingly high.

There was a statistically significant positive correlation between all three instruments as indicated in Table 2.

**Table 2.** Correlations between instruments.

Instruments	Number of observations	Pearson correlation coefficient
AQOL and HUI3	826	0.543*
AQOL and EQ5D	850	0.539*
HUI3 and EQ5D	878	0.551*

* Correlation is significant at the *p* < 0.01 level (two-tailed).

In Table 3, when a *t*-test of the instruments by HIV status was done, it was also found that HIV positive workers score significantly lower on the HUI-3 (*M* = 0.821) as compared to HIV negative people (*M* = 0.914) (*t* = 3.25, *p* < 0.001). This relationship does not hold for HIV positive people on the AQOL (*M* = 0.841) as compared to HIV negative people (*M* = 0.858) (*t* = 1.56, *p* < 0.001) but the difference is significant. In the case of the EQ-5D, HIV positive people (*M* = 0.9186) have a higher quality of life as compared to HIV negative people (*M* = 0.9153) (*t* = 1.56, *p* < 0.001). The difference is minimal but the difference is significant. This result is perplexing.

**Table 3.** Test for significant difference in instruments by HIV status.

	*N*	Mean	Standard deviation	Standard error
AQOL positive	46	0.841	0.156	0.023
AQOL negative	965	0.858	0.163	0.005
EQ5D positive	47	0.918	0.144	0.021
EQ5D negative	1007	0.915	0.173	0.005
HUI3 positive	47	0.821	0.324	0.047
HUI3 negative	1047	0.914	0.168	0.005

When a correlation of age and instrument was undertaken, it was found that younger people score lower on the AQOL measure (*r* = −0.130, *p* < 0.001) but there was no significant finding by age for HUI3 or EQ5D.

When a correlation of nationality and instrument was undertaken (not shown here), it was found that non South Africans score lower on all measures than foreigners but this finding was not significant for each group in any of the three instruments.

## Discussion

The aim of this study was to assess the HR-QOL of diamond miners in conjunction with a VCT programme for HIV. The dual hypothesis underpinning this study was that the HR-QOL of all HIV positive miners would be lower regardless of the instrument used. To this end, three instruments were administered to all miners. Results from these instruments showed that HIV status as a predictor of HR-QOL for diamond miners varied between instruments.

Behavioural characteristics and demographic characteristics were variables of interest and were assessed in addition to overall HR-QOL. Little in the way of statistically significant results were found between HIV status and age, employment status, race, nationality, job band, or race. These findings are surprising in the context of South Africa where race, in particular, and employment status to a lesser extent are usual predictors of HIV status.

Explanations for the surprising HR-QOL results centre on:

(1) The health management programme that the company has implemented meant that all employees are subjected to rigorous annual check-ups, with underground workers subject to bi-annual check-ups. Further, the provision of free ART to all HIV positive workers has been company policy since 2005.
(2) The seemingly inoculating effect that formal employment has on the transmission of HIV in the workplace in the southern African setting (Beard *et al.* 2009).

A review of the South African literature on HIV and AIDS and quality of life reveals a wide range of estimates. Although the prevailing consensus in those studies is that being HIV positive leads to lower quality of life scores. Hughes *et al.* (2004), Jelsma and Ferguson (2004), and Jelsma *et al.* (2005) found that HR-QOL in AIDS patients in various South Africa settings was lower. Hughes *et al.* (2004) found that HR-QOL is severely compromised in late-stage AIDS patients. Peltzer and Phaswana-Mafuya (2008) in their study using the World Health Organization QOL-HIV measure found that a positive HIV status correlated with a lower overall quality of life.

Miners *et al.* (1999) using the SF-36 and EQ-5D questionnaires tested for HR-QOL in haemophiliacs. However, results from both questionnaires showed that HIV status was not a strong predictor of HR-QOL in this patient group. The main reason for this result is that the two HR-QOL instruments may not be sufficiently sensitive to detect clinical details that are important to individuals infected with HIV. Friend – du Preez and Peltzer (2009) investigated the relationship between current symptom status (no symptoms vs. symptoms present) and dimensions of HR-QOL and overall quality of life (poor vs. good) of 612 PHIV, just prior to initiating highly active ART at three public hospitals in South Africa. Physical health and independence were important predictors of higher QOL for patients both with and without symptoms.

Louwagie *et al.* (2007) estimated HR-QOL using the EQ-5D instrument. They reported that those patients waiting to start ART treatment commonly reported health problems. They reported mean HR-QOL weights of 0.69 for patients awaiting ART, while the weight for those on ART was significantly better at 0.80. Beard *et al.* (2009) in a meta-analysis of HIV and HR-QOL studies concluded that HIV positive people generally experience poorer quality of life than HIV negative people and that the provision of ART to HIV positive people results in dramatic improvements in their quality of life.

## Study limitations

There are several limitations of this study. These limitations mainly relate to sample sizes of the various cohorts of workers. Many HIV positive employees, who knew their status, did not participate in the VCT campaign and were therefore unavailable for the study. In terms of the HIV negative cohort, only slightly over 40% of employees who participated in the VCT campaign completed the quality of life questionnaire. This was a problem but not as severe as the HIV positive worker self-selection problem. The participation patterns in the VCT campaign and completion of the quality of life questionnaire by workers highlight the major problem confronting mine management. Many of those workers who know their HIV status as being positive did not participate in the VCT campaign. This outcome would not be an issue if the subsequent number of workers on wellness programmes and enrolled in ART programmes were a high percentage of those eligible. However, this is not the case.

The ease of completion of the three utility instruments varied with the complexity of the instrument. In the HIV-workers, the most complex, the AQOL, with 20 questions by 5 dimensions had the lowest completion rate 990/1163 or 85%, the EQ-5D at 87% and the HUI-3 was highest at 91%.

## Conclusions

Two main conclusions from this measurement of quality of life in a South African diamond mine can be drawn. The first is that the self-reported health of workers both HIV positive and negative is generally high. The second was that the quality of life for HIV positive workers was not lower than HIV negative workers as expected. The results were surprising with HIV positive workers having a lower quality of life as measured by the HUI3, but no difference using the AQOL and the confounding result with the EQ-5D where HIV positive people have a higher quality of life than HIV negative people. This latter conclusion is unexpected. These results suggest that the HR-QOL of HIV positive miners are not uniformly worse than their HIV negative counterparts, although there was not consistency in this result despite statistically significant correlations between the instruments.
